# Anthropometric Indices and Markers of Atherothrombotic Risk in Subjects with Primary Hyperparathyroidism

**DOI:** 10.3390/metabo16030166

**Published:** 2026-02-28

**Authors:** Anda Mihaela Naciu, Eleonora Sargentini, Marco Bravi, Annunziata Nusca, Francesco Grigioni, Luigi Bonifazi Meffe, Nicola Napoli, Andrea Palermo, Gaia Tabacco

**Affiliations:** 1 Unit of Metabolic Bone and Thyroid Disorders, Fondazione Policlinico Universitario Campus Bio-Medico, 00128 Rome, Italy; 2 Unit of Rehabilitation, Fondazione Policlinico Universitario Campus Bio-Medico, 00128 Rome, Italy; 3 Unit of Cardiac Sciences, Department of Medicine, Campus Bio-Medico University, 00128 Rome, Italy; 4 Cardiology Unit, Fondazione Policlinico Universitario Campus Bio-Medico of Rome, 00128 Rome, Italy; 5 Unit of Endocrinology and Diabetes, Campus Bio-Medico University, 00128 Rome, Italy; 6 Department for the Promotion of Human Science and Quality of Life, San Raffaele Open University, 00166 Rome, Italy

**Keywords:** hyperparathyroidism, PTH, hypoparathyroidism, anthropometric parameters, atherothrombotic risk

## Abstract

**Background**: Both primary hyperparathyroidism (PHPT) and chronic hypoparathyroidism (HypoPT) are associated with the onset and development of cardiovascular diseases (CVDs). In particular, PHPT is accompanied by the presence of elevated atherothrombotic risk, while the importance of traditional and new anthropometric indices to reflect the cardiovascular risk remains uncertain in this condition. This study aims to investigate whether novel and traditional anthropometric indices distinguish PHPT and whether these indices are correlated with atherothrombotic risk. **Methods**: A total of 40 subjects with HypoPT, 40 with PHPT and 40 age- and sex-matched control subjects were consecutively enrolled for the evaluation of flow-mediated vasodilation (FMD) and carotid intima–media thickness (IMT). A blood sample was collected for evaluation of calcium–phosphate metabolism, PTH, TSH and 25-hydroxy vitamin D. Physical examination was performed to obtain traditional anthropometric parameters and derived indices of adiposity and cardiometabolic risk (waist-to-height ratio (WHtR), waist-to-hip ratio (WHR) and conicity index (CI)). **Results**: The PHPT group showed higher central adiposity indices (WHtR *p* = 0.002, and CI *p* = 0.008). Among patients with parathyroid disorders, PHPT subjects displayed the highest reduction in FMD (*p* < 0.001) and a marked increase in IMT (*p* < 0.001). In the Ctrl group, WHtR showed a weak-to-moderate positive association with IMT (r = 0.381, *p* = 0.018). In the PHPT group, no anthropometric index was significantly correlated with IMT or FMD (all *p* > 0.05). **Conclusions**: WHtR and CI provide evidence of increased central fat adiposity in PHPT but do not account for impaired atherothrombotic risk, indicating that anthropometric indices may lack relevance to cardiovascular risk in this condition and emphasizing the importance of a specific assessment profile.

## 1. Introduction

Parathyroid disorders in the adult focus primarily on primary hyperparathyroidism (PHPT), characterized by persistent inappropriate syntheses and release of parathyroid hormone (PTH) causing hypercalcemia, and hypoparathyroidism (HypoPT) which is a rare entity caused by markedly decreased or absent levels of circulating PTH [1,2,3,4,5]. Accumulating evidence points to an enhanced cardiovascular (CV) risk in both disorders, with a particularly relevant meaning of PHPT [6,7,8,9,10,11,12]. Hyperparathyroidism and hypercalcemia are invariably linked to cardiovascular complications by direct and indirect effects on cardiomyocytes, endothelial cells, pancreatic β-cells and adipose tissue with induction of the atherothrombotic environment [13,14,15,16,17].

A number of clinical and experimental studies have associated PHPT with higher rates of hypertension, arrhythmias, metabolic derangements and subclinical vascular injury, thus reinforcing the concept that these patients present an increased cardiovascular risk [16,17]. In this regard, we and others have previously shown that patients with parathyroid disorders—in particular those affected by PHPT—have features consistent with an increased atherothrombotic risk profile, hinting at the possibility that excessive PTH might be involved in the pathogenesis of vascular impairment and atherosclerotic phenomena [18,19]. However, epidemiologic findings are partly conflicting, and study design, population composition and distribution in terms of gender structure, age at diagnosis or serum calcium values may account for variable cardiovascular phenotypes in different cohorts. Moreover, the association of PHPT with obesity is still controversial [20]. It has been observed that patients with PHPT have increased body weight and body mass index (BMI), when compared to controls [20]; it is possible that this occurs due to an action of PTH on lipolysis inhibition in adipose tissue. Nonetheless, high levels of PTH seem not to be always associated with weight gain, and evidence shows a non-linear relationship between BMI/weight and the extent of hypersecretion of PTH that may involve downtime weight loss (probably related to hypercalcemia or malnutrition) or increase in thermogenesis [21,22]. In addition, obesity may impact the clinical effects of PHPT in terms of its metabolic and renal consequences [23].

The evaluation of CV risk is based on the detection of early structural and functional vascular changes. In this regard, carotid intima–media thickness (IMT) [24,25] and flow-mediated dilation (FMD) [26] are well-established and highly reproducible modalities for predicting CV risk. IMT has been validated as a subclinical marker of atherosclerosis and predictors for future CV events, while FMD is a well-known non-invasive indicator of endothelial dysfunction. Notwithstanding their great accuracy, these approaches may not be applicable in routine clinical work or mass screening. Thus, anthropometric indices have been attracting interest as simple, cheap and non-invasive markers with a relationship to vascular risk factor. Of these, BMI, waist circumference (WC), waist-to-height ratio (WHtR) and waist-to-hip ratio (WHR) are more widely accepted in routine clinical practice and have a possible role as early screening tools for CV risk [27]. In recent years, new anthropometric indexes have been introduced to better assess body fat distribution and its association with CV risk. In particular, the conicity index (CI) uses weight, height and abdominal circumference variables to estimate the degree of obesity and fat distribution [28]. To the best of our knowledge, no study has yet directly investigated how these indices reflect CV risk in subjects with parathyroid disorders.

The present study aims to investigate whether novel and traditional anthropometric indices distinguish PHPT and are correlated with markers of atherothrombotic risk.

## 2. Materials and Methods

### 2.1. Study Design

This cross-sectional, single-center study took place at the bone outpatient clinic of the Metabolic Bone and Thyroid Disorders Unit of Fondazione Policlinico Universitario Campus Bio-Medico, Rome between March 2021 and July 2023.

### 2.2. Ethics Approval and Consent to Participate

Our protocol adhered to the Declaration of Helsinki and the International Conference on Harmonization Good Clinical Practice, receiving approval from Campus Bio-Medico University ethics committees (89/20 PAR ComEt CBM from 27 November 2020). All participants granted informed consent, allowing the use of their pseudoanonymized information for data analysis. The corresponding author had full access to all the data in the study and took responsibility for its integrity and data analysis.

### 2.3. Participants

Subjects with post-surgical HypoPT and PHPT were consecutively enrolled. HypoPT was defined as hypocalcemia in the presence of a low or inappropriately normal PTH level at least 12 months after surgery [8]. PHPT was defined as elevated or unsuppressed PTH concentrations and persistently elevated (high) total, albumin-corrected, or ionized serum calcium levels (at least two different determinations) [2]. All participants in the study had previously received oral cholecalciferol to ensure sufficient 25-hydroxy vitamin D (25(OH)D) levels. Post-surgical HypoPT participants were receiving thyroxine treatment and maintained normal TSH levels.

We excluded subjects with any other condition that can affect bone and calcium metabolism, such as familial hypocalciuric hypercalcemia, 25(OH)D < 20 ng/mL (for controls); malabsorption diseases; other diseases known to affect bone metabolism (thyrotoxicosis, bowel diseases, chronic hepatic disease, depression, history of Cushing’s, alcoholism, smoking habit, diabetes, antihypertensive therapy, obesity, eating disorders, rheumatological or hematological diseases, chronic inflammatory disease and autoimmune diseases); and administration of drugs affecting bone and calcium metabolism (thiazide diuretic, lithium, bisphosphonates, significant use of glucocorticoids within the past 2 years or any treatment that could affect calcium metabolism). We also excluded patients with a prior history of cardiovascular disorders, as well as those showing symptoms indicative of atherosclerotic cardiovascular disease and/or classified as having high or very high cardiovascular risk according to the SCORE2 system [29]. Additionally, patients with active malignancies other than low-risk, well-differentiated thyroid cancer were not included in the study cohort.

Control subjects without any impairment of calcium, phosphate and PTH concentration were consecutively recruited based on the above-mentioned exclusion criteria from the outpatient clinic of endocrinology, where they were referred for unrelated diseases (thyroid nodules with euthyroidism). To ensure comparability, we performed age- and BMI-based nearest-neighbor matching using the Euclidean distance metric. For each individual in the pathological groups (HypoPT and PHPT), we identified the control subject with the smallest Euclidean distance in a two-dimensional space defined by age and BMI. Matching was performed without replacement to maintain a 1:1 ratio. After matching, the groups were verified for balance in age and BMI, confirming the effectiveness of the matching procedure.

### 2.4. Procedures

#### 2.4.1. Clinical Evaluation

We evaluated the clinical profile of the whole study population by recording the medical history and reviewing the clinical, laboratory and imaging results already performed on patients. Physical examination was performed in all subjects. To minimize selection and measurement bias, all field investigators underwent standardized training before data collection. Weight was measured with a precision biomedical scale and the height was determined with a Harpenden stadiometer. All measures were carried out with participants wearing light clothing and no shoes. A non-elastic tape was used to assess the hip circumference (HC), neck circumference (NC), wrist circumference (CWrist) and waist circumference (WC). Novel and traditional anthropometric indices were calculated using the formulas reported below. BMI = weight (kg)/height^2^(m), cut-off: ≥30 kg/m^2^ [27]; WHR = WC (cm)/HC (cm), cut-off: >0.8 (women) [30]; WHtR = WC (cm)/height (cm), cut-off: >0.5 [31]; conicity index = 0.109^−1^ WC (m) [weight (kg)/height (m)]^−1/2^, cut-off: >1.28 [32].

#### 2.4.2. Biochemical Analysis

Fasting blood sampling was obtained in the morning (from 8:00 to 9:00 AM). Serum total calcium (normal, 8.4–10.2 mg/dL) and albumin were measured using automated methods, and calcium values were corrected for albumin concentration. Serum thyroid stimulating hormone (TSH), blood glucose levels, lipid levels, ionized serum calcium, serum phosphate, 25(OH)D and creatinine were also measured by automated techniques. Intact PTH was assessed by an immunochemiluminometric assay using the automatic analyzer Modular E170 (Roche Diagnostics, Indianapolis, IN, USA). Normal serum iPTH levels ranged between 13 and 85 pg/mL.

#### 2.4.3. Blood Sample Collection

After an 8 h fasting, the peripheral venous blood samples were collected in sterile vacutainer tubes (Vacutainer Systems, Belliver Industrial Estate), which differed according to the analyses to be performed. Blood samples were drawn in tubes with or without anticoagulant (3.2% sodium citrate) to obtain serum and plasma, respectively. Then, they were centrifuged at 300× *g* for 10 min at room temperature, with maximum deceleration (Beckman Coulter-Mod: AllegraX-12R). Serum and plasma samples were stored at −80 °C to analyze the different markers.

#### 2.4.4. Flow-Mediated Vasodilation

FMD was measured in accordance with recent expert consensus [26]. Subjects were not allowed to consume alcohol, caffeine or tobacco or to eat for at least 6 h before the examinations. Also, all vasoactive agents were discontinued for a minimum of 48 h prior to the evaluation, and interrupting nitrate agents, angiotensin-converting-enzyme inhibitors, angiotensin receptor blockers or calcium channel blockers were not administered. Measurements were made in a silent room under temperature control after subjects rested supine for at least 10–15 min. The baseline BAs were measured by selecting a straight arterial segment 8–10 mm above the antecubital fossa with a high-frequency 10 MHz linear-array transducer. Reactive hyperemia was subsequently provoked by inflating a pneumatic cuff positioned on the upper arm to 50 mmHg above systolic blood pressure for 5 min, which was immediately thereafter deflated. A 2nd longitudinal image scan was collected 1 min after cuff release to establish post-occlusion arterial diameter. Five consecutive heart beats per scan were averaged. FMD was calculated as the percent change from baseline diameter and quantified using this formula: FMD = [(highest diameter − baseline diameter)/baseline diameter] × 100. Intra-observer variability was 1.3 ± 0.4%, evaluated in 15 randomly selected subjects.

#### 2.4.5. Carotid Intima–Media Thickness

Bilateral carotid arteries were assessed by an experienced operator with a high-resolution B-mode ultrasound system fitted with a 10 MHz linear-array transducer [33]. The patients were scanned in the supine position, with head slightly tilted and extended and contralateral rotated towards the side to be investigated in order to best visualize cervical vessels. Once the carotid bifurcation was localized in transverse plane, the probe was rotated 90° to generate longitudinal images of common carotid artery, carotid bulb and internal carotid artery.

Carotid IMT was measured in the far wall of both common carotid arteries just proximal to the bifurcation in a segment free from plaque for at least 10 mm on each side. IMT was defined as the distance between the lumen–intima and media–adventitia interfaces. Statistical analysis was performed using the average IMT values of right and left carotid arteries.

#### 2.4.6. Statistical Analysis

Continuous variables were summarized as mean and standard deviation (SD). Cases with missing values in the grouping variable were excluded from group-based analyses using listwise deletion. Between-group differences in anthropometric indices were tested using one-way analysis of variance (ANOVA; Type III sums of squares). Homogeneity of variances was assessed with Levene’s test. When the ANOVA was significant, post hoc pairwise comparisons were performed using Tukey’s correction; effect sizes were reported as η^2^ for ANOVA tests and Cohen’s d for pairwise comparisons; confidence intervals for mean differences were Tukey-adjusted, whereas confidence intervals for Cohen’s d were Bonferroni-adjusted. Pearson’s correlation coefficient (r) was used to quantify associations between anthropometric indices and vascular outcomes (IMT and FMD × 100). The strength of correlation was interpreted based on the absolute value of r using the following guidelines: 0.00–0.19 “very weak”; 0.20–0.39 “weak”; 0.40–0.59 “moderate”; 0.60–0.79 “strong”; 0.80–1.00 “very strong” [34]. In addition, multivariable linear regression models were fitted to estimate independent associations with IMT and FMD × 100, entering group as a categorical predictor (Ctrl as reference) and anthropometric indices as continuous predictors; to assess whether the association between WHtR and vascular markers differed by group, we fitted additional regression models including Group × WHtR interaction terms (control group as reference); multicollinearity was assessed using tolerance and variance inflation factors (VIFs). All tests were two-tailed, and statistical significance was set at α = 0.05. All analysis were performed using JASP Version 0.95.4 (JASP Team (2025)).

## 3. Results

### 3.1. Clinical and Biochemical Features

The study population included 120 adults (40 subjects with HypoPT, 40 with PHPT and 40 controls) (mean age 54.0 ± 11.0 years; 87% female). All demographic, clinical and laboratory data are reported in Table 1. There were no differences between the three groups regarding age, sex or BMI. In the HypoPT group, the mean duration of disease was 11.4 ± 8.5 years. Systolic and diastolic blood pressure, fasting glucose, C-reactive protein, and lipid profile did not significantly differ among controls, HypoPT and PHPT (all *p* > 0.05). The PHPT group showed higher central adiposity indices (WHtR and CI). FMD values (expressed as FMD × 100) notably decreased in patients with PHPT, with a significant difference reported between PHPT and HypoPT groups and controls (*p* < 0.001 between PHPT and controls and *p* = 0.001 between HypoPT and controls) (Table 1). Regarding IMT, patients with both PHPT or HypoPT showed higher values of IMT compared to control subjects (*p* < 0.001 and *p* = 0.001, respectively). Full descriptive statistics are provided in Table 1.

### 3.2. Anthropometric Parameters and Derived Indices of Adiposity and Cardiometabolic Risk

One-way ANOVA showed no statistically significant between-group differences for WC (F (2,116) = 2.786, *p* = 0.066, η^2^ = 0.046), HC (F(2,116) = 2.225, *p* = 0.113, η^2^ = 0.037), NC (F(2,116) = 1.195, *p* = 0.306, η^2^ = 0.020), CWrist (F(2,116) = 0.798, *p* = 0.453, η^2^ = 0.014) or WHR (F(2,116) = 1.369, *p* = 0.258, η^2^ = 0.023).

**Figure 1:**
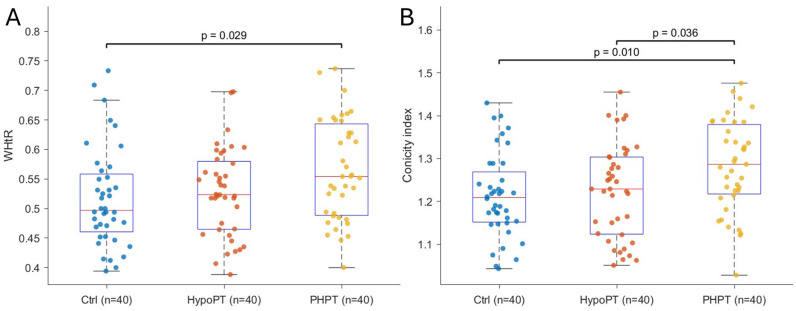
Between-group differences in central adiposity indices. (**A**) shows the distribution of waist-to-height ratio (WHtR) across groups. (**B**) shows the distribution of the conicity index across the same groups. Points represent individual participants; boxplots display the median and interquartile range (IQR), with whiskers indicating 1.5 × IQR. Horizontal brackets denote statistically significant Tukey post hoc comparisons.

Homogeneity of variances was supported for these models (Levene’s tests *p* > 0.05). Conversely, WHtR differed significantly across groups (F (2,116) = 3.671, *p* = 0.028, η^2^ = 0.060) and post hoc comparisons indicated that PHPT exhibited higher WHtR than Ctrl (mean difference = 0.047, 95% CI 0.004 to 0.091; *p* = 0.029; d = 0.58, Bonferroni-adjusted 95% CI 0.03 to 1.14) Figure 1A, whereas the remaining pairwise contrasts were not significant (all *p* > 0.05). Similarly, the CI differed across groups (F (2,116) = 5.072, *p* = 0.008, η^2^ = 0.080). Post hoc tests showed that PHPT had higher CI values compared with Ctrl (mean difference = 0.070, 95% CI 0.014 to 0.125; *p* = 0.010; d = 0.67, Bonferroni-adjusted 95% CI 0.11 to 1.22) and HypoPT (mean difference = 0.059, 95% CI 0.003 to 0.115; *p* = 0.036; d = 0.56, Bonferroni-adjusted 95% CI 0.01 to 1.12) Figure 1B, while Ctrl and HypoPT did not differ (*p* = 0.890). All analyses are reported in Table 2. In interaction models, WHtR remained independently associated with IMT (B = 1.428, *p* = 0.007). The WHtR × Group interaction was not significant for HypoPT (*p* = 0.848), but was for PHPT (B = −1.131, *p* = 0.051). For FMD × 100, WHtR was not associated (*p* = 0.357) and neither interaction term was significant (*p* = 0.145 and *p* = 0.645) (Table S1).

**Figure 2:**
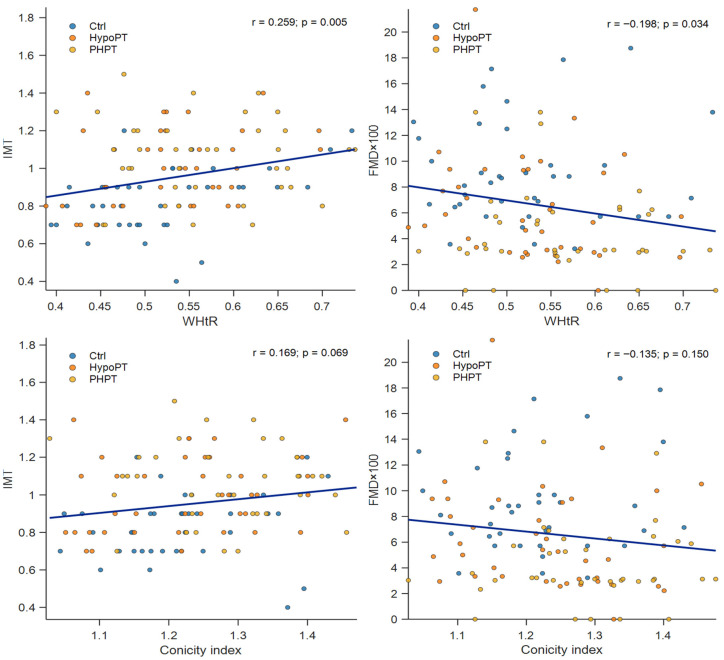
Correlations between central adiposity indices and vascular outcomes. Scatterplots show the relationships between waist-to-height ratio (WHtR) and conicity index with intima–media thickness (IMT) (**left panels**) and flow-mediated dilation (FMD × 100) (**right panels**). Points represent individual participants and are color-coded by group (Ctrl, HypoPT, PHPT). The solid line represents the overall least-squares regression fit. Pearson’s correlation coefficients (r) and associated *p* values are reported in each panel.

Anthropometric indices and their correlations with vascular outcomes were generally very weak to weak and differed by subgroup. In the Ctrl group, WHtR showed a weak-to-moderate positive association with IMT (r = 0.381, *p* = 0.018), while WC and HC displayed weak positive trends with IMT that did not reach statistical significance (WC: r = 0.300, *p* = 0.067; HC: r = 0.304, *p* = 0.063). No significant correlations with FMD× 100 were observed in Ctrl. In HypoPT, WHR was moderately positively correlated with IMT (r = 0.372, *p* = 0.020), whereas WHtR showed a weak and non-significant association with IMT (r = 0.301, *p* = 0.062). Associations with FMD × 100 were non-significant, although HC and WHtR showed weak inverse trends (HC: r = −0.274, *p* = 0.091; WHtR: r = −0.237, *p* = 0.146). In the PHPT group, no anthropometric index was significantly correlated with IMT or FMD × 100 (all *p* > 0.05). The largest (non-significant) association was observed between CWrist and FMD × 100 (r = −0.310, *p* = 0.058). Overall, subgroup-specific analyses suggest that the strength and direction of the associations between central adiposity measures and vascular outcomes may vary across groups; complete correlation coefficients are reported in Table 3, while Figure 2 illustrates scatterplots for WHtR and CI.

Two multivariable linear regression models were fitted to examine independent associations of anthropometric indices with IMT and FMD × 100, adjusting for Group (Ctrl as reference) and other anthropometric covariates (WHtR, CI, NC, CWrist, WHR). Multicollinearity was low (all VIFs ≤ 2.29). For IMT as dependent variable the overall model was significant (F(7,108) = 4.917, *p* < 0.001) and explained 24.2% of the variance in IMT (R^2^ = 0.242; adjusted R^2^ = 0.193; RMSE = 0.209; N = 116). WHtR was independently associated with higher IMT (B = 0.940, SE = 0.423, β = 0.335, *p* = 0.028). Compared with Ctrl, both patient groups exhibited higher IMT after adjustment (HypoPT: B = 0.184, SE = 0.048, *p* < 0.001; PHPT: B = 0.224, SE = 0.050, *p* < 0.001). CI, NC, CWrist and WHR were not significant predictors (all *p* > 0.05). For FMD × 100 as dependent variable, the overall model was significant (F(7,107) = 4.806, *p* < 0.001) and explained 23.9% of the variance in FMD × 100 (R^2^ = 0.239; adjusted R^2^ = 0.189; RMSE = 3.825; N = 115). After adjustment, both groups showed lower FMD × 100 than Ctrl (HypoPT: B = −2.924, SE = 0.875, *p* = 0.001; PHPT: B = −4.488, SE = 0.917, *p* < 0.001). WHtR showed a non-significant inverse association with FMD × 100 (B = −13.706, SE = 8.178, β = −0.269, *p* = 0.097), while CI, NC, CWrist, and WHR were not significant (all *p* > 0.05). Table 4 report multivariable linear regression models.

## 4. Discussion

The current study was conducted to assess whether novel and traditional anthropometric indices distinguish PHPT and if they are correlated with markers of atherothrombotic risk. The main results could be summarized as follows: (i) indexes of central adiposity relating to body size such as WHtR and conicity index were significantly different between patients with PHPT and either controls or HypoPT subjects; (ii) classical anthropometric indexes, WHR, did not distinguish among groups; and (iii) WHtR and CI did not correlate with IMT/FMD within the PHPT group; however, in the pooled adjusted regression across the whole cohort, WHtR was independently associated with IMT. In the interaction analyses, the WHtR × Group term was not significant for HypoPT, while it was borderline for PHPT, suggesting a possible attenuation of the WHtR–IMT association in PHPT compared with controls. For FMD × 100, neither WHtR nor Group × WHtR interaction terms were significant (Table S1).

Anthropometric indices have been widely used as surrogates for cardiometabolic risk because of the very strong association that exists between central adiposity and atherosclerosis in the general population [35,36]. In the control group of our study, this paradigm was evidenced by the significant positive association of WHtR with IMT that confirmed central adiposity indices as valid markers for subclinical atherosclerosis, even in a healthy population. In HypoPT patients, WHR was also the only anthropometric parameter that showed a significant correlation with IMT, hence indicating that relative fat distribution can still be related to vascular health in the absence of PTH excess. The relatively long duration of HypoPT in our cohort (mean 11.4 years) may have influenced vascular parameters; however, the cross-sectional design did not allow evaluation of temporal effects. Notably, when considering the cohort as a whole, the multivariable regression analysis (adjusted for group and other covariates) indicated an independent association between WHtR and IMT, highlighting an overall link between central adiposity and vascular structure at the population level. By contrast, within the PHPT subgroup, correlations between central adiposity indices (WHtR and conicity index) and IMT/FMD were weak and not statistically significant, despite a distinct anthropometric profile characterized by higher WHtR and conicity index, indicative of preferential central fat accumulation relative to body size. This pattern suggests that PHPT is linked to a specific distribution of body fat, potentially reflecting metabolic consequences of chronic PTH excess, including changes in insulin sensitivity, lipid metabolism, and energy homeostasis [23]. Nevertheless, the absence of association between these indices and IMT and/or FMD suggests that in this disorder central adiposity may not simply reflect the major determinant of vascular injury.

This discrepancy in the association between the anthropometric indicators and vascular measures among PHPT patients may be explained by the direct effect on vessels of PTH [18]. It has been well established, based on experimental and clinical evidence, that PTH has pleiotropic effects on the vascular wall, involving endothelial dysfunction, enhanced oxidative stress, VSMC proliferation, and medial calcification [37,38]. These processes could, in turn, be involved in arterial remodeling and atherosclerotic burden, irrespective of the distribution of body fat [37]. While prior studies suggest that parathyroidectomy may improve vascular structure and function in PHPT—particularly in more overt disease—evidence remains heterogeneous [39,40,41,42]. Thus, the weak associations observed between anthropometric indices and vascular parameters in PHPT may suggest that vascular alterations in this condition are not fully explained by adiposity alone. It is possible that hormonal factors related to parathyroid dysfunction contribute to vascular changes; however, given the cross-sectional design of the study, causal inferences cannot be made.

In addition, IMT is a structural sequelae of cumulative vascular injury, whereas FMD constitutes dynamic endothelial function, which is affected by several factors including inflammation, mineral metabolism, calcium–phosphate homeostasis and nitric oxide bioavailability. In PHPT subjects, these mechanisms may take precedence over the contribution of adiposity-related systems, resulting in a lack of association between anthropometric parameters and vascular outcomes. The lack of correlation to FMD in any group and also the consistency of inverse trends within PHPT patients, thus reinforce the idea that endothelial dysfunction may result from non-anthropometric pathways in this condition.

Collectively, these results indicate that the CV relevance of anthropometric indicators is dependent on context- and disease-related factors. Major cardiovascular risk factors were comparable among groups, and overt cardiometabolic comorbidities were excluded; however, residual confounding cannot be completely excluded. Although WHtR and CI are useful for early detection of modified fat distribution in PHPT patients, their efficacy might not be enough to determine the multifaceted atherothrombotic risk in this group. In contrast to the general population in which central adiposity is one of the main determinants of vascular damage, PHPT seems to be characterized by a dissociation between body fat distribution and vascular injury, possibly reflecting additional disease-specific mechanisms.

From a clinical point of view, these findings address the limitation of considering only anthropometric indices when assessing cardiovascular risk in diseases of endocrine organs with direct hormonal effects on the vessels. In PHPT, cardiovascular risk evaluation might be not complete without considering hormonal, inflammatory and mineral metabolism-related aspects in addition to classic anthropometric variables. Furthermore, future research may evaluate potential associations between anthropometric indices and elastographic features of parathyroid adenomas [43].

A major strength of our study is the careful exclusion of subjects with overt cardiometabolic comorbidities, which allowed us to reduce confounding and better isolate the relationship between parathyroid status, anthropometric indices and vascular markers. However, this also represents a limitation, as the study population is not fully representative of the broader PHPT population, in whom cardiovascular risk factors are common. Therefore, our findings should be interpreted primarily as mechanistic insights rather than directly generalizable to routine clinical settings.

Several limitations should be acknowledged. Owing to the cross-sectional nature of the study, causal inferences cannot be made. No formal a priori sample size calculation was performed for correlation or regression analyses. With 40 participants per subgroup, the study had approximately 80% power to detect correlations of about r = 0.47 or greater (α = 0.05, two-sided), indicating that smaller associations may not have been detected. Therefore, non-significant findings should be interpreted cautiously, and larger studies are warranted to confirm these results. The predominance of female participants reflects the known epidemiology of PHPT; however, sex-related differences in cardiovascular risk may limit the generalizability of the findings to male patients. Furthermore, analyses of visceral adiposity using direct imaging were not available. Despite this, the agreement of results across a range of anthropometric indices and vascular outcomes may lend robustness to our findings.

In conclusion, WHtR and conicity index may be sensitive measures of changed central adiposity in hyperparathyroid individuals, but in this cohort they showed limited and inconsistent associations with markers of vascular structure and function. While these findings suggest that traditional anthropometric indices may not fully capture cardiovascular risk in PHPT, modest associations cannot be excluded, and larger studies are warranted to better define their clinical utility. Dysrelation between cardiovascular risk and anthropometric indices indicates that, in PHPT, these may not be the best markers of cardiovascular risk factors as previously found in the general population; this highlights the importance of looking for a disease-specific approach to assessing cardiovascular risk. Further comprehensive and prospective studies with long-term follow-up are required to show the clinical reflection of the study and the correlation between biochemical and clinical parameters.

## Figures and Tables

**Table 1:** Descriptive characteristics of the study sample by group. Values are mean (SD).

Variable	Ctrl (*n* = 40)	HypoPT (*n* = 40)	PHPT (*n* = 40)	*p* for Trend
Age [years]	52.50 (10.60)	53.52 (12.30)	56.67 (8.23)	0.185
Weight [kg]	69.44 (13.44)	74.60 (16.86)	72.13 (15.16)	0.373
Height [m]	1.66 (0.07)	1.68 (0.07)	1.64 (0.08)	0.148
BMI [kg/m^2^]	25.30 (4.77)	26.43 (5.24)	26.73 (5.64)	0.500
Calcium (mg/dL)	9.51 (0.33)	8.53 (0.57)	10.78 (0.55)	<0.001
Phosphate (mg/dL)	3.22 (0.48)	4.05 (0.89)	2.62 (0.62)	<0.001
PTH (pg/mL)	64.58 (22.55)	13.15 (10.47)	185.87 (100.53)	<0.001
TSH (mlU/L)	1.43 (0.73)	1.87 (1.6)	2.1 (3.97)	0.872
WC [cm]	85.60 (13.01)	88.69 (13.69)	92.70 (13.51)	0.066
HC [cm]	101.53 (10.48)	104.66 (11.15)	107.08 (13.42)	0.113
NC [cm]	33.64 (3.44)	34.16 (4.31)	34.96 (3.69)	0.306
Cwrist [cm]	15.70 (1.60)	15.96 (1.95)	16.26 (2.27)	0.453
WHR	0.84 (0.07)	0.84 (0.07)	0.86 (0.07)	0.258
WHtR	0.52 (0.08)	0.53 (0.07)	0.56 (0.09)	0.0028
Conicity index	1.21 (0.10)	1.22 (0.11)	1.28 (0.11)	0.008
IMT [mm]	0.81 (0.22)	1.00 (0.20)	1.05 (0.21)	<0.001
FMD × 100 [%]	9.12 (4.01)	6.20 (4.03)	4.58 (3.44)	<0.001

Note: WC, waist circumference; HC, hip circumference; NC, neck circumference; WHR, waist-to-hip ratio; WHtR, waist-to-height ratio; IMT, intima–media thickness; FMD × 100, flow-mediated dilation expressed as percentage × 100. *p* from one-way ANOVA.

**Table 2:** Anthropometric indices differences across groups. One-way ANOVA and Tukey HSD pairwise comparisons of anthropometric indices across groups.

Outcome	F (2,116)	*p* (ANOVA)	η^2^	Ctrl vs. HypoPT *p* †	Ctrl vs. PHPT *p* †	HypoPT vs. PHPT *p* †
WC	2.79	0.066	0.046	0.560	0.052	0.381
HC	2.23	0.113	0.037	0.458	0.094	0.632
NC	1.20	0.306	0.020	0.813	0.278	0.624
CWrist	0.80	0.453	0.014	0.821	0.419	0.783
WHR	1.37	0.258	0.023	0.957	0.269	0.412
WHtR	3.67	**0.028**	0.060	0.826	**0.029**	0.116
Conicity index	5.07	**0.008**	0.080	0.890	**0.010**	**0.036**

† Tukey HSD multiplicity-adjusted *p*-values for the family of three pairwise comparisons. WC, waist circumference; HC, hip circumference; NC, neck circumference; WHR, waist-to-hip ratio; WHtR, waist-to-height ratio. In bold *p* < 0.05. One-way ANOVA (F, *p*) and Tukey HSD pairwise *p*-values.

**Table 3:** Pearson correlations between anthropometric indices and vascular outcomes. Values are reported as r (*p*-value).

Anthropometric Variable	IMT			FMD × 100		
	Ctrl	HypoPT	PHPT	Ctrl	HypoPT	PHPT
WC	0.300 (0.067)	0.287 (0.077)	−0.016 (0.921)	0.137 (0.413)	−0.202 (0.219)	−0.174 (0.295)
HC	0.304 (0.063)	0.123 (0.457)	0.115 (0.487)	0.019 (0.912)	−0.274 (0.091)	−0.156 (0.349)
NC	0.029 (0.864)	0.165 (0.316)	0.147 (0.372)	0.288 (0.080)	−0.059 (0.720)	−0.210 (0.206)
CWrist	0.057 (0.735)	0.119 (0.469)	−0.023 (0.891)	0.109 (0.513)	0.047 (0.775)	−0.310 (0.058)
WHR	0.152 (0.361)	0.372 (0.020)	−0.241 (0.140)	0.222 (0.180)	−0.024 (0.887)	−0.079 (0.637)
WHtR	0.381 (0.018)	0.301 (0.062)	−0.054 (0.746)	0.011 (0.948)	−0.237 (0.146)	−0.157 (0.348)
Conicity index	0.165 (0.322)	0.257 (0.114)	−0.035 (0.835)	0.161 (0.334)	−0.188 (0.250)	−0.135 (0.150)

Note. Strength categories are based on the absolute value of r: 0.00–0.19 “very weak”; 0.20–0.39 “weak”; 0.40–0.59 “moderate”; 0.60–0.79 “strong”; 0.80–1.00 “very strong”. WC, waist circumference; HC, hip circumference; NC, neck circumference; CWrist, wrist circumference; WHR, waist-to-hip ratio; WHtR, waist-to-height ratio; IMT, intima–media thickness; FMD × 100, flow-mediated dilation expressed as percentage × 100.

**Table 4:** Multivariable linear regression models for IMT and FMD × 100. Values are unstandardized coefficients B (SE); β denotes standardized coefficients (continuous predictors only). Reference category for Group is Ctrl.

Predictor	IMT			FMD × 100		
	B (SE)	β	*p*	B (SE)	β	*p*
Intercept	0.789 (0.287)	—	0.007	5.140 (5.364)	—	0.340
WHtR	0.940 (0.423)	0.335	0.028	−13.706 (8.178)	−0.269	0.097
Conicity index	−0.425 (0.377)	−0.196	0.262	1.940 (7.673)	0.049	0.801
Wrist circumference	−0.012 (0.013)	−0.104	0.360	−0.079 (0.243)	−0.037	0.747
WHR	0.224 (0.469)	0.067	0.633	6.715 (8.924)	0.111	0.453
NC	0.002 (0.007)	0.027	0.826	0.129 (0.135)	0.118	0.342
Group: HypoPT (vs. Ctrl)	0.184 (0.048)	—	<0.001	−2.924 (0.875)	—	0.001
Group: PHPT (vs. Ctrl)	0.224 (0.050)	—	<0.001	−4.488 (0.917)	—	<0.001

IMT model fit: N = 116; R^2^ = 0.242; adjusted R^2^ = 0.193; RMSE = 0.209; F(7,108) = 4.917; *p* < 0.001; FMD × 100 model fit: N = 115; R^2^ = 0.239; adjusted R^2^ = 0.189; RMSE = 3.825; F(7,107) = 4.806; *p* < 0.001. Abbreviations: NC, neck circumference; WHR, waist-to-hip ratio; WHtR, waist-to-height ratio; IMT, intima–media thickness; FMD × 100, flow-mediated dilation expressed as percentage × 100.

## Data Availability

Some or all datasets generated and/or analyzed during the current study are not publicly available, but are available from the corresponding author on reasonable request.

## Supplementary Materials

The following supporting information can be downloaded at: https://www.mdpi.com/article/10.3390/metabo16030166/s1, Table S1: Multivariable linear regression models for IMT and FMD × 100 including Group × WHtR interaction terms.
