# Supplementary material for: Anthropometric Indices and Markers of Atherothrombotic Risk in Subjects with Primary Hyperparathyroidism
Source: Metabolites. 2026 Feb 28;16(3):166. doi: 10.3390/metabo16030166 (PMC13027883; doi:10.3390/metabo16030166)
Supplement: Supplementary file 1 [file metabolites-16-00166-s001.zip › metabolites-4102783-supplementary.pdf]

metabolites

MDPI

Table S1. Multivariable linear regression models for IMT and FMD×100 including Group × WHtR interaction terms.

Predictor	IMT			FMD×100		
	B (SE)	β	p	B (SE)	β	p
(Intercept)	0.618 (0.34)	—	0.072	0.4 (6.448)	—	0.951
WHtR	1.428 (0.519)	0.509	0.007	−9.124 (9.87)	−0.179	0.357
Conicity index	−0.380 (0.373)	−0.176	0.311	2.916 (7.698)	0.073	0.706
Cwrist	−0.011 (0.013)	−0.092	0.410	−0.061 (0.243)	−0.028	0.803
WHR	0.051 (0.47)	0.015	0.914	6.847 (9.023)	0.113	0.450
NC	0.001 (0.007)	0.021	0.865	0.152 (0.136)	0.139	0.267
Group (HypoPT)†	0.243 (0.325)	—	0.456	5.854 (6.038)	—	0.335
Group (PHPT)†	0.842 (0.314)	—	0.008	−2.041 (5.81)	—	0.726
WHtR × Group (HypoPT)†	−0.117 (0.612)	—	0.848	−16.772 (11.422)	—	0.145
WHtR × Group (PHPT)†	−1.131 (0.572)	—	0.051	−4.893 (10.583)	—	0.645

Model fit (IMT): N = 116; R^2 = 0.273; adjusted R^2 = 0.211; RMSE = 0.207; F(9,106) = 4.427; p < 0.001. Model fit (FMD×100): N = 115; R^2 = 0.255; adjusted R^2 = 0.191; RMSE = 3.821; F(9,105) = 3.992; p < 0.001. Abbreviations: NC, neck circumference; WHR, waist-to-hip ratio; WHtR, waist-to-height ratio; IMT, intima–media thickness; FMD×100, flow-mediated dilation expressed as percentage ×100. †Control group is the reference category. Interaction terms represent the difference in WHtR slope vs Controls.
